# The Rapid Identification and Evaluation of the Resonant Noise of a Cooling Module Based on the Frequency Difference Sensitivity Method

**DOI:** 10.3390/s23239568

**Published:** 2023-12-02

**Authors:** Yinhui Zhong, Yinong Li

**Affiliations:** College of Mechanical and Vehicle Engineering, Chongqing University, Chongqing 400044, China; zhongyh123456@126.com

**Keywords:** modal frequency, frequency difference sensitivity method, identification and evaluation, cooling module

## Abstract

The energy amplification factor transmitted from the excitation source to the response end cannot be identified quickly and accurately using the method of obtaining modal frequency combined with damping through modal frequency resonance. As a result, the above method cannot be used to further evaluate the effect of structural improvement. In this paper, a frequency difference sensitivity method is proposed in order to improve the efficiency of the above identification and evaluation processes while also guaranteeing accuracy. Firstly, a theoretical model suitable for the damping and rigid-body-mode frequency range of the cooling module is established according to the frequency difference between the excitation frequency, .system mode frequency, and vibration response sensitivity. Then, the accuracy and effect of the model are studied from the perspective of a simulation and experiment. The model can be used to identify and evaluate the resonance problem of the vehicle cooling module quickly and accurately. The energy amplification factor of the fan to the passive end of the cooling module can be efficiently predicted using the frequency difference sensitivity method proposed in this paper while ensuring accuracy. The quantized noise-reduction effect of the interior before and after the improvement can be efficiently predicted and evaluated using said model. The literature shows that the resonance of a vehicle cooling module can be identified and evaluated quickly and accurately via the frequency difference sensitivity method, which can overcome the problems that the conventional modal frequency resonance method encounters in identifying and evaluating quickly.

## 1. Introduction

Fan noise is an important source of noise that produces noise in the interior of vehicles. The noise of automotive axial fans can generally be divided into structural and air sound. Structural sound mainly comes from the vibration of the motor and the vibration caused by the aerodynamic force of the fan blade during the operation of the fan [1]. Generally, the structural noise generated by the excitation force from the fan’s imbalance and cooling module resonance is the worst in idle or low-speed conditions [2].

In order to solve this resonance problem, some scholars have studied the influential factors of modal frequency. It has been pointed out that natural frequency has minimal effect with changes in viscosity but decreases with increases in fluid medium density when using the hammer method [3]. For vehicle track systems, the length, stiffness, and applied unsprung mass of the track have an influence on the resonance frequency of vehicle track systems. When the orbit is longer than a critical value, the difference in the natural frequency of the system is not significant. With an increase in stiffness, the natural frequency increases slightly. As the unsprung mass increases, the natural frequency decreases slightly [4].

In order to solve the resonance problem, some scholars have carried out relevant research to determine a method of solving the resonance problem. You Taiwen [5] mentioned that the excitation frequency is close to the modal frequency of the floor, resulting in the local resonance of the floor, and local stiffness optimization can better solve the resonance problem. While the deviation between the gear engagement frequency and the natural frequency is larger, the resonance amplitude become smaller [6]. As the damping ratio increases, the natural frequency decreases slightly [7], and the vibration peak decreases accordingly [8,9]. For free vibrations, the logarithmic attenuation method or the Hilbert transform method are commonly used to identify the damping ratio [10].

In order to solve the cooling module resonance problem, some scholars have conducted research on the improvement scheme and effect. At present, there are two solutions to the problem of cooling module resonance noise caused by axial flow fans in vehicles. On the one hand, the centrifugal force generated by fan rotation is reduced from the aspect of controlling the source. On the other hand, the vibration isolation pad, body sheet metal, bracket, cavity mode, etc. are improved in terms of improving the transmission paths [11]. The rigid body mode of the automobile cooling module resonates with the second-order excitation frequency of the engine, which causes noise and vibration inside the vehicle. The structure of the vibration isolation pad of the cooling module is adjusted, and the stiffness is optimized using dividing grids, establishing simulation models for simulation. Sensors and debugging equipment for trials are arranged so as to achieve the effect of frequency avoidance and vibration isolation [12,13,14].

The current conventional identification method uses a frequency ratio and damping to identify the energy amplification factor caused by the resonance when the excitation frequency is close to the modal frequency of the system with the aim of reducing the resonant noise of the cooling module caused by the axial fan of the whole vehicle [15]. However, damping cannot be obtained quickly. The damping ratio is obtained by using the modal frequency and half-power bandwidth method within the conventional method [10]. Then, the energy amplification factor is identified in combination with the conventional frequency ratio vibration isolation theory. Other identification methods need to be obtained using simulations or experiments. Because the finite element simulation analysis method requires the division of grids and various settings [16,17], and the experimental analysis method needs sensors and debugging equipment to be arranged [18,19] (and the resonance characteristics are analyzed via signal processing) [20,21], these methods’ identification efficiencies are low. Finite element simulation analyses and experimental analyses are also used in evaluations of the noise reduction effect, and their efficiencies are also low. Therefore, a resonant noise identification and evaluation method for a vehicle cooling module is proposed in this paper, and is based on frequency difference sensitivity method, which can quickly identify the energy amplification factor when a fan’s excitation frequency is close to the system’s modal frequency. Sequentially, the causes are analyzed, and the structural parameters are improved more efficiently. Then, the improved quantitative noise reduction effect can be quickly calculated and predicted.

## 2. Noise Identification and Evaluation Theory Based on Frequency Difference Sensitivity Method

When the excitation frequency is coupled with the modal frequency of the system component, the modal frequency and damping are generally obtained through modal frequency resonance so as to identify the energy amplification factor transmitted by the excitation source to the response end.

It is difficult to separately judge the energy superposition of the components in the noise source and transmission path accurately and quickly. This is due to the superposition of multiple physical fields and multiple sound fields in complex working conditions such as vehicle idling with air conditioning. Thus, improved noise quantization effects cannot be accurately reflected. The frequency ratio is usually taken as the conventional method of analyzing the vibration isolation rate, which can be abstracted as a single-degree-of-freedom forced vibration. The absolute value of the ratio between the amplitude of the force on the vehicle body and the amplitude of the exciting force makes up the transfer rate under the action of simple harmonic excitation force, which can be expressed as follows:

(1) $$T=\left|\frac{1+j2\xi \lambda }{1-\lambda^{2}+j2\xi \lambda }\right|=\sqrt{\frac{1+{\left(2\xi \lambda \right)}^{2}}{{\left(1-\lambda^{2}\right)}^{2}+{\left(2\xi \lambda \right)}^{2}}}$$

where ξ is the material damping ratio, and λ is the frequency ratio, that is, the ratio of the excitation force frequency to the natural frequency. λ can be expressed as $f/f_{n}$.

**Figure 1:**
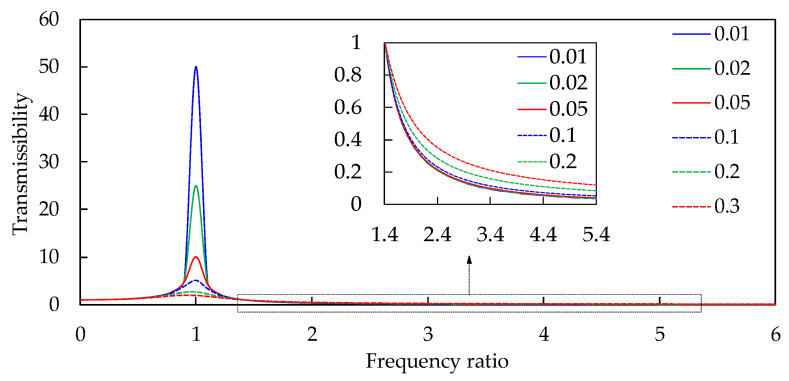
Transmission curve of vibration isolation system.

According to Equation (1), the curve of the transmission rate’s variance with the frequency ratio can be obtained, as shown in Figure 1. This group of curves can be roughly divided into three regions. The first region is controlled by the elastic force; the second region is the damping region, and near the position λ=1, the system is prone to resonance; and finally, the third region is the vibration reduction region, and the system’s vibration is controlled by inertial force. In this area, the frequency ratio is higher, and the vibration isolation effect is better. 

The natural frequency under damped conditions is

(2) $$f_{n}=\frac{\sqrt{\left(1-\xi^{2}\right)k}}{2\pi \sqrt{m}}=\frac{1}{2\pi }\sqrt{\frac{k}{m}-\frac{c^{2}}{4m^{2}}}$$

where c is the material damping coefficient, and *m* represents the quality of the cooling module, which is generally about 10 kg.

The excitation frequency, the natural frequency, and the damping of the system components are obtained. Then, the transmission from the excitation point to the passive end of the body, namely the energy amplification factor, can be effectively analyzed using this method. However, the damping of the system is generally difficult to determine, and the damping ratio can be obtained via two methods. The first is a modal resonance curve combined with the half-power bandwidth method, and the second is composed of relevant bench experiments on rubber blocks; both of these methods are very inefficient. Therefore, a new method is proposed for identification and improvement using the frequency difference sensitivity method based on the frequency ratio theory in this paper. The energy amplification factor transmitted to the passive end can be identified quickly and accurately via this method when the cooling module resonates with the fan excitation. In this way, the effect of internal quantization noise reduction can be obtained before and after the improvement. The frequency difference sensitivity method can be studied from two perspectives: the identification and evaluation of the resonant noise of a vehicle cooling module.

### 2.1. Noise Identification Based on the Frequency Difference Sensitivity Method

The expression of the damping coefficient and hysteresis angle of the rubber vibration isolation pad is as follows:

(3) $$c=\frac{K\sin\varphi }{\omega_{n}}$$

where c is the damping coefficient, K is the dynamic stiffness, φ is the phase angle, and $\omega_{n}$ is the natural circular frequency.

The phase angle of the rubber vibration isolation pad of the cooling module and the other parts is generally between 3° and 5°, and its change is very small, usually 4°. The dynamic stiffness of the rubber block of the cooling module is generally 100 N/mm, and the modal frequency is generally about 30 Hz. According to Formula (3), the damping coefficient c=0.037. This value is subsequently used, and the frequency difference sensitivity method of noise recognition is further analyzed in this paper.

The frequency difference sensitivity method includes the calculation of the frequency difference and frequency difference sensitivity (it contains damping information; the damping coefficient change is very small, so the impact of damping change is not analyzed). This method of analysis is more efficient, effective, and intuitive. When the sensitivity is higher, the frequency difference become smaller, and the response become the greater; thus, this discrimination index can be expressed as

(4) $$y=aS-b{\Delta f}^{2}$$

where Δf represents the frequency difference between the excitation frequency f and the natural frequency $f_{n}$ of the component in the transmission path, S represents the sensitivity under different frequency differences, and a and b are undetermined coefficients. In order to give the formula more generality, the function y, sensitivity S, and variable Δf are normalized. 

Sensitivity is defined as follows:

(5) $$S=\frac{dy}{df}=\frac{\Delta y}{\Delta f}$$

Therefore, Formula (4) can be changed to

(6) $$y=a\frac{dy}{df}-b\Delta f^{2}$$

Thereby, Equation (6) is solved to obtain

(7) $$y=-b\Delta f^{2}-2ab\Delta f-2a^{2}b+ce^{\frac{\Delta f}{a}}$$

where a, b, and c are undetermined coefficients.

**Figure 2:**
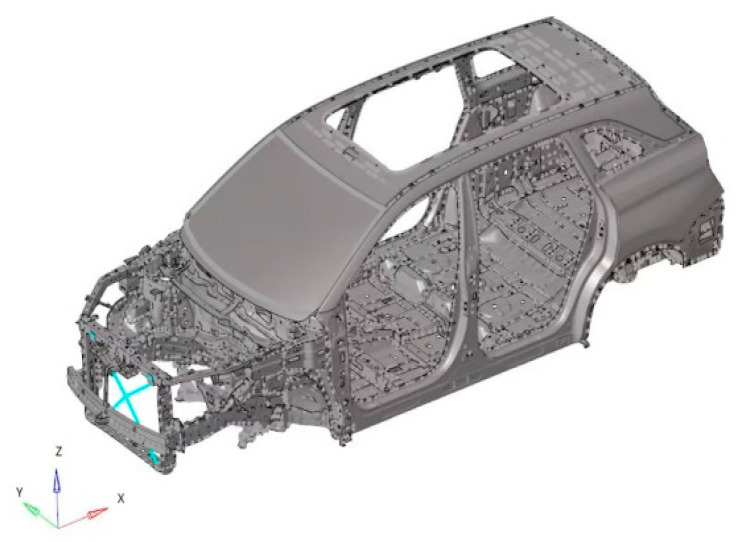
Sweeping frequency loading of the cooling module with vehicle coordinates. The intersection of the blue lines represents where the force is applied.

**Figure 3:**
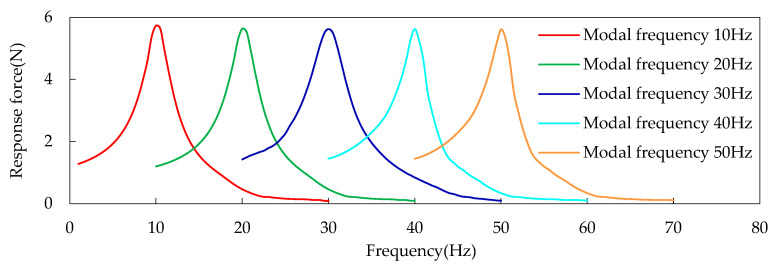
Response force varying with frequency difference curves.

In order to determine the parameter values corresponding to Equation (7), the center position of the fan is stimulated using the sweeping frequency to obtain the relationship between the force response of the cooling module and the frequency difference. The rigid body modal frequency of the cooling module is generally in the range of 10~50 Hz. A load force of 1 N is applied in the Y direction and Z direction of the fan, as shown in Figure 2. The response of the cooling module at each frequency is obtained by adjusting the stiffness of the vibration isolation pad of the cooling module, as shown in Figure 3.

It can be seen from Figure 3 that the resonance and attenuation characteristics of the installation point of the passive end of the cooling module, with the cooling module mode frequency ranging from 10 Hz to 50 Hz, are similar under the condition that 1 N load force is applied in both the Y and Z directions of the fan. The vibration of the system in this region is controlled and amplified slightly by the elastic force, while the excitation frequency is less than the natural frequency of the system component, which is consistent with the conclusion of the linear mode theory of vibration. Energy amplification and energy attenuation exist in this region, while the excitation frequency is more than or equal to the natural frequency of the system components, which are the main research focus of this paper. When the frequency difference is 20 Hz, the energy amplification factor (representing the energy decay) is about 0.1, thereby fully meeting the requirements of vibration isolation. Thus, 20 Hz is taken as the frequency difference of the entire region.

**Figure 4:**
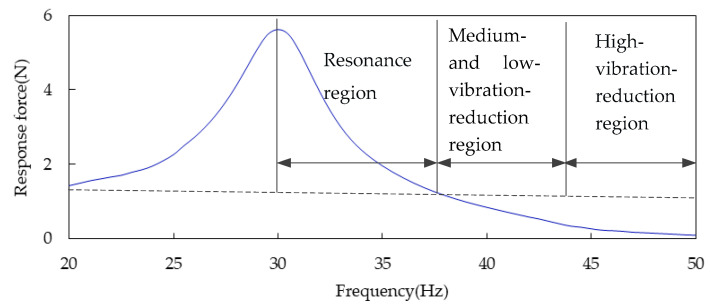
Regional division of the response force with variations in frequency.

The vehicle frequency region is divided into the resonance region and the vibration reduction region in this paper (see Figure 4) according to the traditional criteria (see Figure 1). At the junction point of the two regions, the frequency difference is about 7.5 Hz, and the energy amplification factor is just 1. The characteristic lines of the damping region of the cooling module are similar to the traditional characteristic lines. They begin to decay rapidly with the increase in the frequency difference, and then decay slowly, as shown in Figure 1 and Figure 3. The mathematical model in this paper is a quadratic polynomial with one variable. It is expressed in the frequency band of fast decay and slow decay, respectively, which can improve the prediction accuracy of the theoretical model. The interface frequency between the fast and slow decay regions is about 14.5 Hz. The entire region can be roughly divided into the resonance region, the medium- and low-vibration-reduction region, and the high-vibration-reduction region, as shown in Figure 4. Therefore, the three frequency bands of the cooling module are, respectively [0 Hz, 7.5 Hz], [7.5 Hz, 14.5 Hz], and [14.5 Hz, 20 Hz]. 

**Figure 5:**
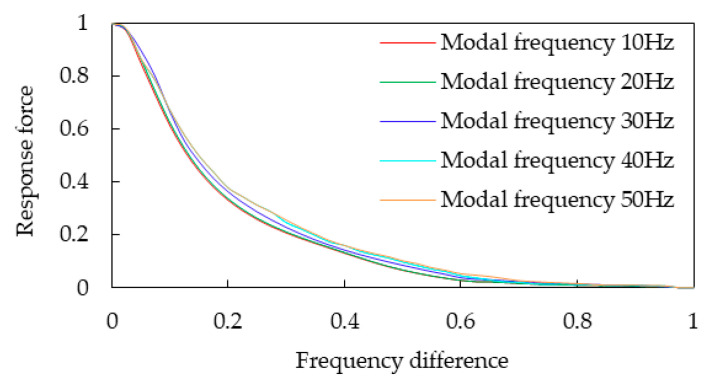
Variation curve of response force with frequency difference after normalization.

The response force, which varies with the frequency difference, is normalized in the range of 20 Hz, where the excitation frequency is higher than or equal to the natural frequency of the system components, as shown in Figure 5.

**Figure 6:**
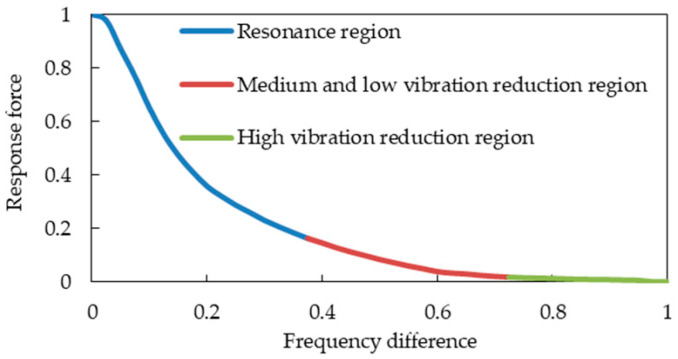
Average value of response force with frequency difference after normalization.

As shown in Figure 5, the curve of the response force with frequency difference after normalization is averaged to obtain the graph shown in Figure 6. This curve can be divided into three regions: the resonance region, the medium- and low-vibration-reduction region, and the high-vibration-reduction region, as shown in Figure 6.

The three regional key points shown in Figure 6 are substituted into Equation (7), and the values of parameters a, b, and c can be obtained by solving nonlinear equations. These three parameters are substituted into Equation (7) to obtain

(8) $$\left\{\begin{array}{cc} y=6.0955\Delta f^{2}-4.615\Delta f+1.052, & 0\leq \Delta f\leq 0.375\\ y=1.0175\Delta f^{2}-1.5361\Delta f+0.5969, & 0.375\leq \Delta f\leq 0.725\\ y=-0.057\Delta f^{2}+0.0389\Delta f+0.0184, & 0.725\leq \Delta f\leq 1 \end{array}\right.$$

where the frequency difference Δf in [0, 0.375] is the resonance region, the frequency difference Δf in [0.375, 0.725] is the low–medium-vibration-reduction region, and the frequency difference Δf in [0.725, 1] is the high-vibration-reduction region.

According to Equation (8), when the response force is 1 N, the energy does not decay, and the index is 0.17. Therefore, the response index is around 0.17 (the dividing limit), while the excitation frequency is 20 Hz higher than the natural frequency; the frequency difference and response are normalized as well. The energy will decay below 0.17, and the energy will also be amplified above 0.17. 

The response index after normalization can be obtained according to Equation (8). The maximum value of the energy amplification factor transmitted by the fan to the body end of the cooling module installation point is 5.6, and the minimum value is 0.1. According to the normalization theory, the actual energy amplification factor can be obtained by the response index multiplying by (5.6 − 0.1) plus 0.1.

The response index can only be related to the frequency difference using this method, meaning the problem of low calculation efficiency caused by damping can be avoided; therefore, the energy amplification factor can be quickly identified when the excitation frequency is close to the cooling module. The accuracy of the theoretical model can be improved by dividing the frequency region into three regions.

### 2.2. Noise Evaluation Based on Frequency Difference Sensitivity Method

As the mode of the cooling module or the fan excitation frequency is changed, the transfer function from the body end of the cooling module to the driver position in the car is unchanged. Therefore, the noise reduction amount in the car can be converted into the amount of improvement in the response value of the body end in order to analyze the quantified noise reduction effect of the car before and after the improvement more intuitively. This is expressed in dB, as follows:

(9) $$E=20log\frac{Y_{1}}{Y_{2}}=20log\frac{(5.6-0.1)y_{1}+0.1}{(5.6-0.1)y_{2}+0.1}=20log\frac{y_{1}+0.0182}{y_{2}+0.0182}$$

where E is the noise reduction before and after improvement, $Y_{1}$ represents the amplification factor of the original state, $Y_{2}$ represents the amplification factor after improvement, $y_{1}$ represents the response value of the original state, and $y_{2}$ represents the response value after improvement. 

Equation (8) is substituted into Equation (9), yielding

(10) $$\left\{\begin{array}{cc} E=20log\frac{6.0955\Delta f_{1}^{2}-4.615\Delta f_{1}+1.0702}{6.0955\Delta f_{2}^{2}-4.615\Delta f_{2}+1.0702}, & 0\leq \Delta f\leq 0.375\\ E=20log\frac{1.0175\Delta f_{1}^{2}-1.5361\Delta f_{1}+0.6151}{1.0175\Delta f_{2}^{2}-1.5361\Delta f_{2}+0.6151}, & 0.375\leq \Delta f\leq 0.725\\ E=20log\frac{-0.057\Delta f_{1}^{2}+0.0389\Delta f_{1}+0.0366}{-0.057\Delta f_{2}^{2}+0.0389\Delta f_{2}+0.0366}, & 0.725\leq \Delta f\leq 1 \end{array}\right.$$

where $\Delta f_{1}$ is the difference between the original excitation frequency and the system mode frequency, and $\Delta f_{2}$ is the difference between the improved excitation frequency and the system mode frequency. 

Equation (10) is processed by weighting A so that it is consistent with the perception of the human ear in the car. The difference in the linear sound pressure level $S_{L}$ is converted into the difference in the sound pressure level $S_{A}$ after weighting A. The difference between the A-weighted sound pressure level $S_{A}$ and the linear sound pressure level $S_{L}$ at a certain problem frequency f via the analysis of the test data of the LMS equipment (Leuven test system) is as follows:

(11) $$S_{A}-S_{L}=2+20log\frac{{12,200}^{2}f^{4}}{\sqrt{\left(f^{2}+107.7^{2}\right)\left(f^{2}+737.9^{2}\right)}\left(f^{2}+20.6^{2}\right)\left(f^{2}+{12,200}^{2}\right)}$$

The system’s modal frequency before and after the improvement is unchanged; the excitation frequency is adjusted to avoid resonance frequency in order to obtain the system mode resonance caused by the excitation frequency. Thereby, the noise reduction is not affected by the weight of A, and Equation (10) still applies. 

The system’s modal frequency is adjusted to avoid the frequency of the system’s modal resonance caused by excitation frequency; the excitation frequency is unchanged. Thus, the system’s modal frequency changes before and after the improvement, and the noise reduction is thereby affected by the weight of A:

(12) $$\begin{array}{cc} E & =20log\frac{{12,200}^{2}f_{n1}^{4}}{\sqrt{\left(f_{n1}^{2}+107.7^{2}\right)\left(f_{n1}^{2}+737.9^{2}\right)}\left(f_{n1}^{2}+20.6^{2}\right)\left(f_{n1}^{2}+{12,200}^{2}\right)}\\ & -20log\frac{{12,200}^{2}f_{n2}^{4}}{\sqrt{\left(f_{n2}^{2}+107.7^{2}\right)\left(f_{n2}^{2}+737.9^{2}\right)}\left(f_{n2}^{2}+20.6^{2}\right)\left(f_{n2}^{2}+{12,200}^{2}\right)}\\ & +\left\{\begin{array}{cc} 20log\frac{6.0955\Delta f_{1}^{2}-4.615\Delta f_{1}+1.0702}{6.0955\Delta f_{2}^{2}-4.615\Delta f_{2}+1.0702}, & 0\leq \Delta f\leq 0.375\\ 20log\frac{1.0175\Delta f_{1}^{2}-1.5361\Delta f_{1}+0.6151}{1.0175\Delta f_{2}^{2}-1.5361\Delta f_{2}+0.6151}, & 0.375\leq \Delta f\leq 0.725\\ 20log\frac{-0.057\Delta f_{1}^{2}+0.0389\Delta f_{1}+0.0366}{-0.057\Delta f_{2}^{2}+0.0389\Delta f_{2}+0.0366}, & 0.725\leq \Delta f\leq 1 \end{array}\right. \end{array}$$

where $f_{n1}$ is the modal frequency of the original system, and $f_{n2}$ is the modal frequency of the improved new system. 

As long as the excitation frequency f and natural frequency $f_{n}$ are known, the frequency difference $\Delta f=f-f_{n}$ can be obtained. Then, the energy amplification factor transmitted by the fan to the body end of the cooling module can be quickly obtained through Formula (8), which is both intuitive and practical. Then, the quantization noise reduction effect before and after improvement can be more intuitively obtained through Equation (10) or Equation (12). The noise is treated with the weight of A, which is more consistent with subjective perceptions. 

This method also avoids the influence of damping. As long as the excitation frequency is known, and the modal frequency before and after optimization is optimized, the noise reduction effect of the vehicle can be converted into the force reduction effect of the body end of the cooling module, which significantly improves efficiency in comparison with traditional simulations and tests. The accuracy of the theoretical model can be improved by dividing the frequency region into three regions.

The frequency difference can be obtained in reverse to reduce the sensitivity of frequency difference in order to achieve the target of noise reduction; this provides a basis for the design of the structural parameters of frequency avoidance. 

## 3. Noise Identification and Evaluation Accuracy Based on the Frequency Difference Sensitivity Method

The resonant noise of the cooling module is identified and evaluated from the perspective of simulations and trials in order to verify the identification accuracy of the frequency difference sensitivity method and the prediction accuracy of the improved scheme.

### 3.1. Simulation Analysis

**Figure 7:**
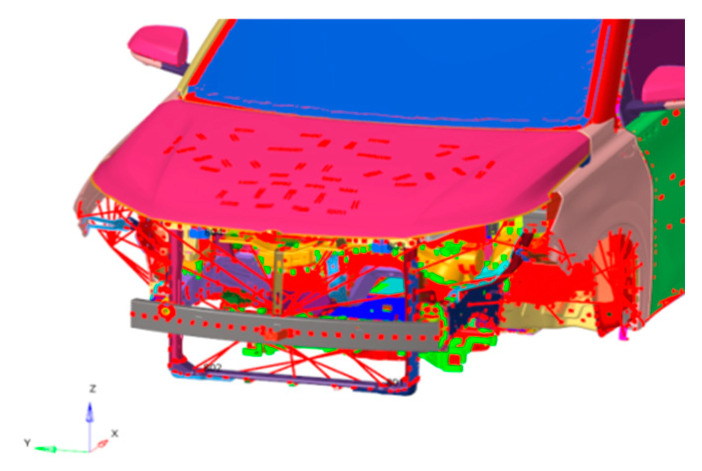
Grid model of the TB body cooling module structure.

**Figure 8:**
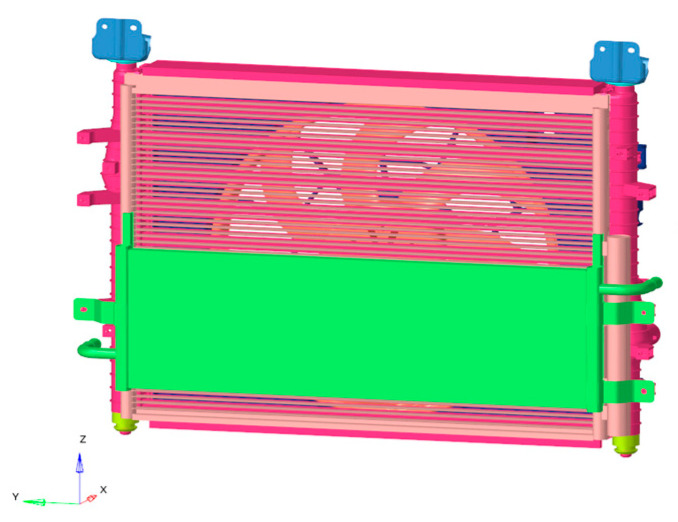
Grid model of the cooling module structure.

Because the modal response of low frequency is studied in this study, the TB body can be used for simulation calculations. Firstly, the TB body and cooling module are grid-divided, as shown in Figure 7 and Figure 8. The fan blade is rotated at 2400 r/m, so the fan’s excitation frequency is 40 Hz, and an unbalance of 30 g·mm is used as the input condition. 

**Figure 9:**
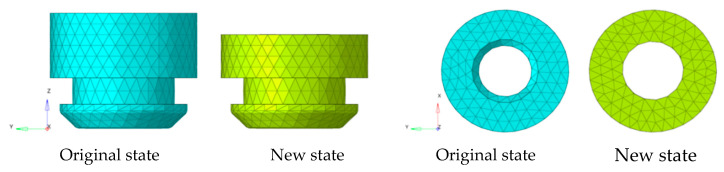
Front and top view of bottom vibration isolation pad.

**Figure 10:**
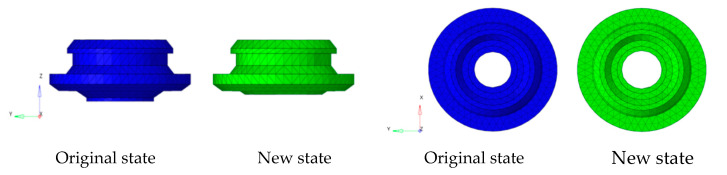
Front and top view of top vibration isolation pad.

The modal frequency includes the damping coefficient and stiffness information from Equation (3), so the degree of influence of the different damping coefficients and modal frequencies on the theoretical model in this study can be expressed using the modal frequency. The vibration isolation pad installed on the bottom right (representing the right installation point on the bottom of the cooling module), bottom left, top right, and top left are sequentially cut off by a third on the basis of the existing cooling module, as shown in Figure 9 and Figure 10. The new state indicates that one third is cut off from different directions. Since the excitation frequency is unchanged, different modal frequencies are simulated using the above method, and then various frequency differences (the difference between the excitation frequency and the modal frequency) and response forces are obtained. The response forces correspond to various frequency differences and are obtained via normalization processing in the same manner; moreover, the theoretical response forces calculated by Equation (8) under the same conditions of frequency difference are compared. Then, the deviation of the theoretical response force compared with simulations under the same frequency difference conditions is verified.

**Figure 11:**
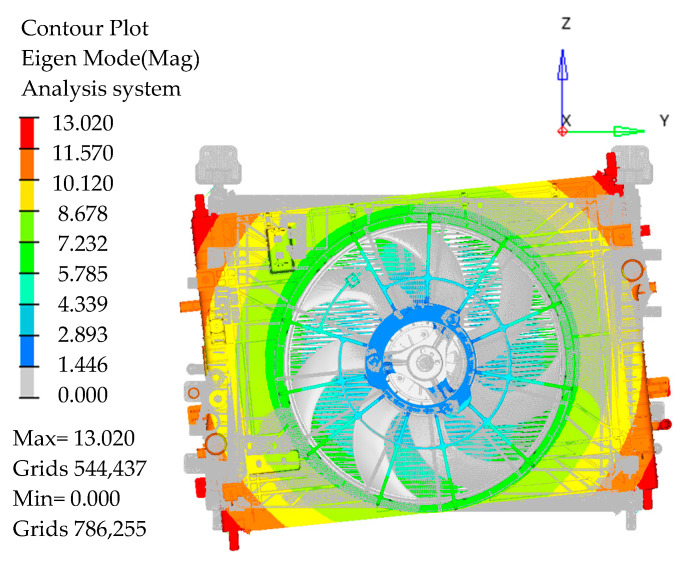
Mode shapes of 40 Hz around the *X*-axis.

**Figure 12:**
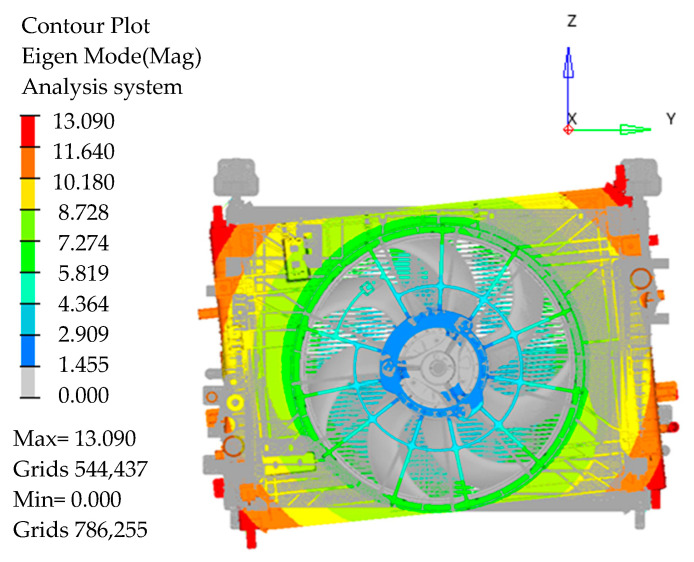
Mode shapes of 38.5 Hz around the *X*-axis.

The modal frequencies of the existing cooling module and the cooling module with one third of the bottom right vibration isolation pad cut off are, respectively, 40 Hz and 38.5 Hz in this paper, as shown in Figure 11 and Figure 12.

### 3.2. Trial Verification

**Figure 13:**
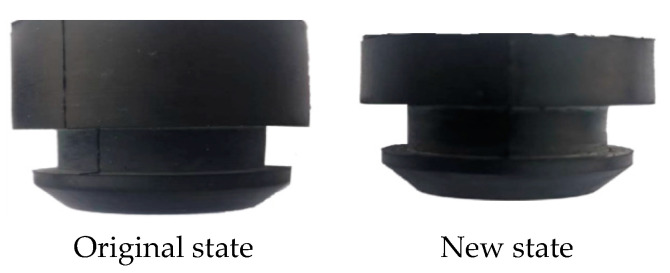
Bottom vibration isolation pad. (Suggestion: Reservation).

**Figure 14:**
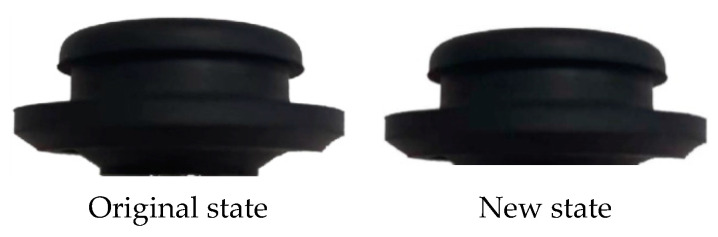
Top vibration isolation pad. (Suggestion: Reservation).

One third of the bottom right vibration isolation pad of the cooling module is cut off based on the original state used for verifying the accuracy of the theory and simulation model, as shown in Figure 13 and Figure 14.

#### 3.2.1. Method of Obtaining Exciting Force

The exciting force generated by the fan can be obtained using Newton’s second law. A trial analysis was carried out in three steps in the anechoic chamber. 

**Figure 15:**
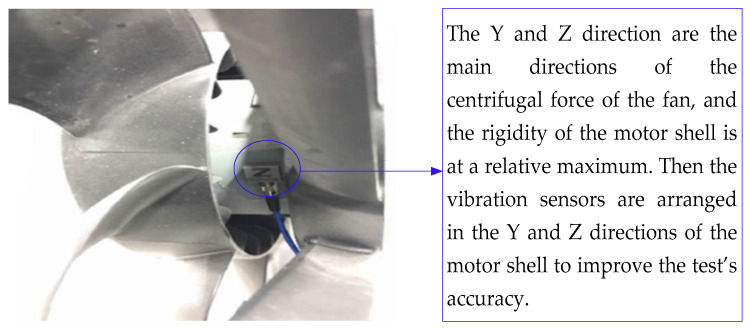
Vibration sensor layout in the motor shell.

① A vibration sensor was arranged in the horizontal direction (Y direction) of the fan motor shell, as shown in Figure 15. Similarly, a vibration sensor was arranged in the vertical direction (Z direction) of the motor shell.

② The whole vehicle was placed in the anechoic chamber. The external voltage regulator power supply was connected. The fan speed was maintained at 2400 r/min. Then, the acceleration *a* of the motor shell surface was tested. 

③ The mass *m* of the fan was weighed out, and the exciting force generated by the fan was found to be *F* = *ma*. 

The acceleration as measured by the horizontal and vertical sensors of the fan motor was averaged in each direction in order to improve the accuracy of the exciting force.

#### 3.2.2. Acquisition Method of Response Force

There are four installation points at the passive end of the cooling module. There are three directions from each installation point. Thus, there are 12 transmission paths and 12 exciting forces at the installation point of the body end, which are the response forces of the cooling module. The response forces are obtained using the inverse matrix method. The testing analysis is carried out in three steps in the anechoic chamber. 

① The whole vehicle was put in the anechoic chamber. The external voltage regulating power supply was connected. The fan speed was maintained at 2400 r/min. Then, the sound pressure in the driver’s right ear and the acceleration of each reference degree of freedom were tested. 

**Figure 16:**
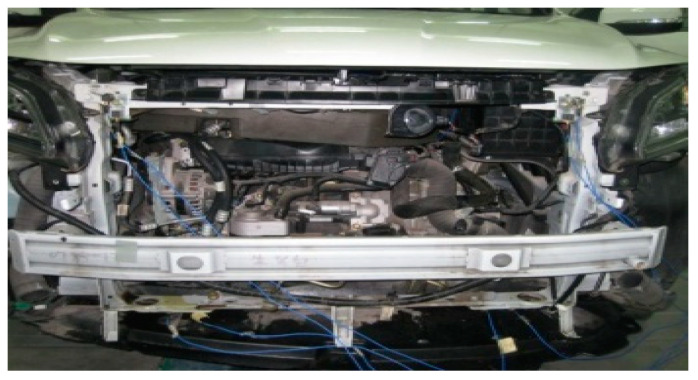
Trial scene after removing the cooling module.

**Figure 17:**
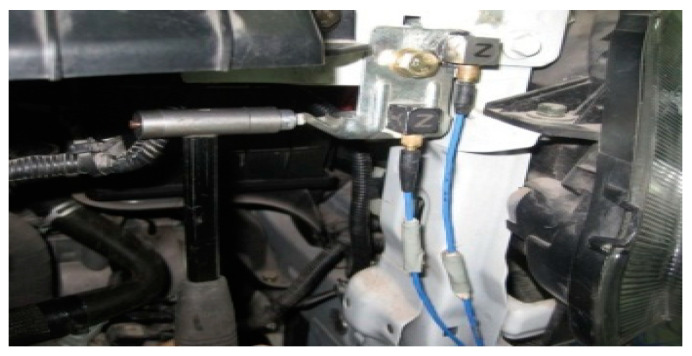
Measuring transfer function using the hammering method.

② The cooling module was removed to decouple the exciting force of each body mounting point, and the trial scene is shown in Figure 16. A force hammer was used to hit the body mounting point from the X, Y, and Z directions to obtain the transfer function of each body mounting point to the corresponding reference degree of freedom acceleration response. The bracket is rigidly connected to the body, and two vibration sensors are arranged at bracket of the top left mounting point to improve accuracy, which is shown in Figure 17.

③ The matrix inversion method [10] was adopted to obtain the coupling response force of the joint point of the body end, comprehensively considering the convenience and accuracy in this study, which can be expressed as follows:

(13) $$\left[\begin{array}{c} F_{1}\\ F_{2}\\ \vdots \\ F_{12} \end{array}\right]={\left[\begin{array}{cccc} H_{1,1} & H_{1,2} & \cdots & H_{1,12}\\ H_{2,1} & H_{2,2} & \ldots & H_{2,12}\\ \vdots & \vdots & \ddots & \vdots \\ H_{24,1} & H_{24,2} & \ldots & H_{24,15} \end{array}\right]}^{-1}\times \left[\begin{array}{c} a_{1}\\ a_{2}\\ \vdots \\ a_{24} \end{array}\right]$$

where *H*_1,1_, *H*_1,2_, ⋯, *H*_24,12_ are the transfer functions of input force *F* to response *a*. 

There are 12 forces at the coupled response points and 24 response vectors at the reference points, which can be directly obtained via testing. The degree of reference freedom is more than the number of coupled response forces, which can be fitted using the least-squares method to improve the accuracy of the response force. On this basis, the response forces of four points in each direction are superimposed, producing the response force of the passive end of the cooling module’s installation point.

### 3.3. Analysis of Noise Identification Accuracy Based on the Frequency Difference Sensitivity Method

A cooling module with a modal frequency of 40 Hz and 38.5 Hz was tested in a real vehicle to verify the theoretical, simulation, and trial deviations of the variation in response force with frequency differences in order to verify the identification accuracy of the frequency difference sensitivity method, as shown in Table 1. It can be seen from Table 1 that the modal frequency of the original state is 40 Hz, while the excitation frequency is 40 Hz; thus, the frequency difference is 0 Hz, and the error between theory and simulation is 5%. The error between the simulation and trial is 0%. The modal frequency is 38.5 Hz after 1/3 of the bottom right vibration isolation pad is cut off. The frequency difference is therefore 1.5 Hz, and the error between theory and simulation is 1.6%. The error between the simulation and experiment is 2.6%, and the accuracy requirements are thus met. The following three schemes (on the basis of the first scheme, in which one third of the bottom left, top right, and top left installation points of the vibration isolation pad are sequentially cut, as shown in Table 2) are verified in accordance with the simulation method for the accuracy of the theoretical model.

Table 2 shows a comparison between the theoretical and simulation results of the variation in response force with frequency difference. It can be seen from Table 2 that the accuracy of the theoretical model is less than 5% within the specified range, which can be used to identify the resonant noise of the cooling module.

### 3.4. Precision Analysis of Noise Evaluation Based on the Frequency Difference Sensitivity Method

The energy amplification factor of the passive end of the cooling module can be identified using the frequency difference sensitivity method, with the aim of solving the problem of energy amplification, which is encountered when frequency of the fan excitation source is close to natural frequency of the cooling module. The quantitative noise reduction effect of the passive end of the cooling module after the improved scheme can be evaluated using the above method as well. Therefore, the evaluation accuracy of the above schemes is further analyzed in this study.

The interior noise after cutting off one third of the bottom right vibration isolation pad of the cooling module is compared to the module in its original state in order to verify the accuracy of the theory and simulation model; the results of said comparison are shown in Table 3. The error between the theory and simulation results is 0.6 dB (A), and that between the simulation and test is 0.2 dB (A), thereby meeting accuracy requirements. Therefore, the following three schemes (on the basis of the first scheme in Table 2, in which one third of the bottom left, top right, and top left mounting points of the vibration isolation pad are sequentially cut) are simulated to verify the evaluation accuracy of the frequency difference sensitivity method.

Table 4 shows the comparison results of interior noise before and after improvement of different improvement schemes. It can be seen from Table 4 that the error of the frequency difference sensitivity method is less than 2 dB (A) (and within the specified range), meaning it can be used to evaluate the resonant noise of the cooling module before and after the improvements induced by the scheme.

## 4. Noise Identification and Evaluation Effect Based on Frequency Difference Sensitivity Method

This method of identifying and evaluating the resonant noise of a vehicle cooling module is very practical. A certain problem is taken as an example, and this method of identifying and evaluating the resonant noise of a vehicle cooling module is applied to solve practical problems in this study.

There is the obvious “roar” in SUV cars when the temperature is above 35 °C and the air conditioner is working, which seriously affects the subjective perception of the comfort of riding in the vehicle. 

When the high-speed fan relay is removed and the high-speed fan stops working, the “roar” disappears. When the low-speed fan relay is removed, the low-speed fan stops working, and the “roar” still exists. Thus, it can be preliminarily judged that this “roar” comes from the high-speed fan.

**Figure 18:**
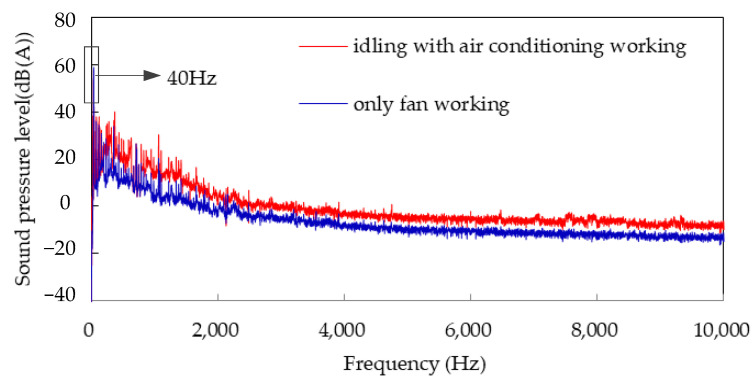
Comparison of noise inside the vehicle when idling with the air conditioning working, and with a single fan operating.

A noise trial was carried out in an anechoic room. The environmental temperature was adjusted to above 35 °C. The test conditions were those of idling with the air conditioning turned on. A single fan maintained at 2400 r/min was tested as the input condition in order to analyze the influence of the fan on the noise inside the car. The fan was powered by an external regulated power supply in order to avoid the interference of the engine. A microphone was positioned at the right ear of the driver, and LMS equipment was used for testing. The results are shown in Figure 18.

The “roar” generated by the air conditioning in the vehicle mainly contributes to the middle- and low-frequency bands; in particular, the peak value at 40 Hz can reach 58.9 dB (A), and the single fan produces 59.2 dB (A), as shown in Figure 18. The noise of the vehicle from the single fan at 40 Hz is slightly louder than that of the air conditioning because of an equipment error. It can be seen that the noise at 40 Hz of the air conditioner running idly is the main contributor to both subjective perceptions and objective test results. Thus, the fan was used as the noise source to study the transmission path.

**Figure 19:**
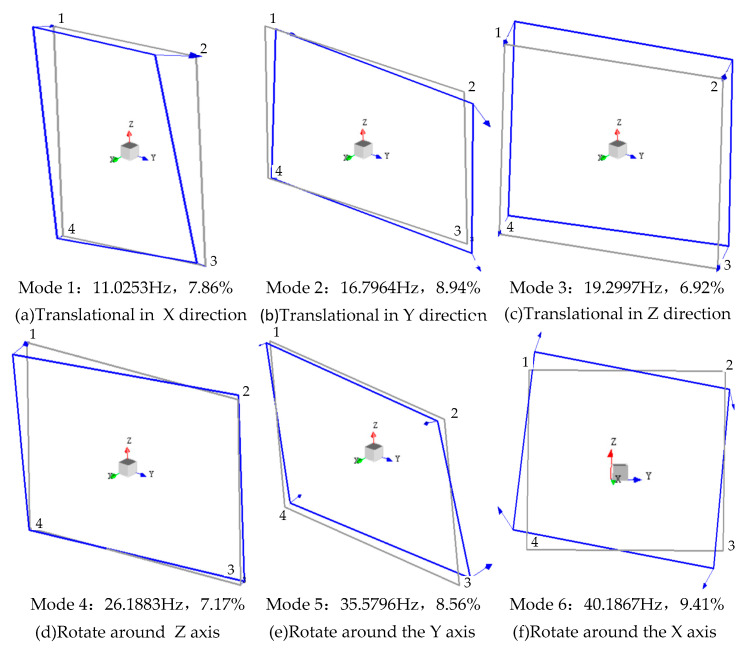
Vibration mode of the cooling module.

The Y direction of each installation point at the passive end of the cooling module contributes much more at 40 Hz than other directions, according to our transmission path analysis. The imbalance of the fan, tested using direct measurements, meets the basic requirements of 30 g·mm. The vibration isolation quantity at 40 Hz in the Y direction of the installation point of the cooling module does not exceed 7 dB, according to the LMS test; however, it is still not certain that the passive energy transmitted by the fan to the installation point of the cooling module is amplified. We found that the modal frequency of the cooling module is 40.2 Hz (through modal testing), and its vibration mode is rotation around the X-axis, as shown in Figure 19.

The modal frequency is obtained through modal frequency resonance. Then, the level of system damping is obtained according to the half-power broadband method. Additionally, then, the energy amplification factor of the fan excitation, transmitted to the cooling module’s passive end, is obtained via the frequency ratio shown in Equation (1). The efficiency of this conventional method is low, and it can be seen from the above that the efficiencies of finite element simulation analysis and experimental analysis are lower. Thus, the frequency difference sensitivity method is adopted in this study. The above modal frequency almost coincides with the excitation frequency; that is, the frequency difference is 0.2 Hz. It can be seen that the index y is 1 according to Equation (8). So, the energy is amplified about five point six times, which seriously exceeds the standard and needs to be improved.

The effect of improving the resonant noise of vehicle cooling modules can be identified and evaluated using the frequency difference sensitivity method. An improved scheme is proposed to predict and evaluate this factor, according to the resonance of the axial flow fan excitation frequency and the coincident modal frequency. This improvement effect cannot be obtained using the conventional frequency ratio method. The finite element simulation analysis method needs the grid to be divided and various conditions to be set. The experimental analysis method needs vibration sensors and debugging equipment to be arranged in order to test the noise reduction effect of the vehicle before and after changes to the scheme. The efficiencies of the above methods are all low.

As increasing the fan speed will increase noise, reducing the speed will affect the air volume; thus, the fan speed remains unchanged. The excitation frequency is maintained, and the modal frequency of the cooling module is reduced via adjusting the dynamic stiffness of cooling module in the Y direction; subsequently, the frequency difference becomes larger, and the frequency difference sensitivity will be reduced.

**Figure 20:**
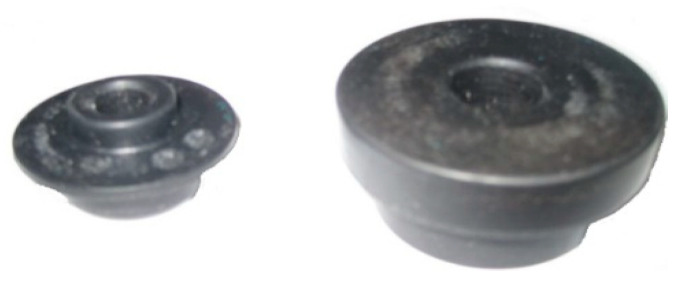
Top and bottom vibration isolator of the cooling module.

The two top vibration isolation pads of the cooling module are the same vibration isolation pads of the cooling module connected to the upper beam, and the two bottom vibration isolation pads are the same vibration isolation pads of the cooling module connected to the lower beam, as shown in Figure 20.

**Figure 21:**
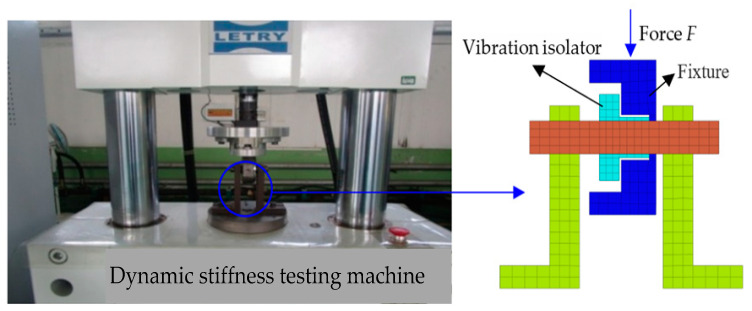
Setup of the dynamic stiffness trial of the vibration isolator.

The dynamic stiffnesses of the four vibration isolation pads of the cooling module are reduced at 40 Hz in order to achieve a better vibration isolation effect, as shown in Figure 21. A schematic diagram of the parts and fixtures shown in the blue circle on the dynamic stiffness test bench after a section inspection is shown in Figure 21. The fixture is made into the following structure shown in blue to protect the integrity of the vibration isolation pad. The force generated by the fixture acts on the radius direction of the vibration isolation pad under the action of force F; the thinner position bears the force in the radius direction, and the thicker position does not bear the force. The vibration isolation pad is not damaged and can continue to be used after the test is completed.

The indicator Y is 0.17, while the natural frequency of cooling module is adjusted to 32.5 Hz. Because 0.17 is the critical index, the excitation energy of the fan is not amplified. The interior noise is reduced by 19 dB (A) after improvement, according to Formula (12), which meets the noise reduction requirements.

The results before and after adjusting the dynamic stiffness at 40 Hz are shown in Table 5. Although the dynamic stiffness of the improved vibration isolation pad is reduced, it still meets the common stiffness requirements of engineering.

**Figure 22:**
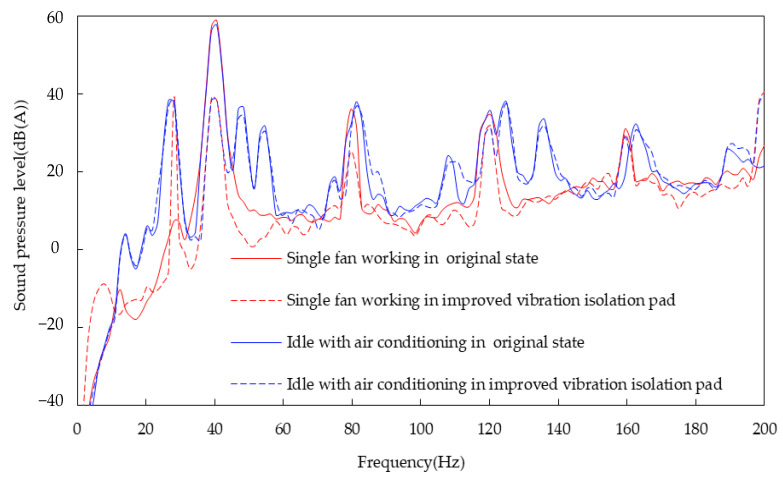
Comparison of noise before and after improvement.

A comparison of the noise spectrum inside the vehicle before and after the improvement of the vibration isolation pad is shown in Figure 22. 

Table 6 is obtained to describe the amount of noise reduction at the key frequencies in Figure 22 in more detail. It can be seen from Figure 22 and Table 6 that the interior noise peak value at the new modal frequency of 32.5 Hz, as calculated by the frequency difference sensitivity method, is 19 dB (A) lower than that at 40 Hz, when only the fan is working. The theoretical error is 0.6 dB (A) lower than that measured, thereby meeting accuracy requirements. At the same time, the peak value of the increase in the noise at the new modal frequency of 32.5 Hz (in idle conditions with the air conditioning working) is also 19.4 dB (A); this is lower than the peak value of 40 Hz. The total sound pressure level is reduced by 3.3 dB (A). The transmission of structural noise can be controlled effectively using this method, and the problem of interior noise caused by fans can be better solved. Since the transmission path of the installation point in the Y direction from the passive end of cooling module was improved (and the interior noise reached an acceptable level), further improvements were not made to other paths.

It can be seen that the conventional frequency ratio method can only use the frequency ratio $\sqrt{2}$ as the critical point to roughly judge whether the energy is amplified when the fan is transmitted to the passive end of the cooling module through the above analysis. The amplification factor cannot be quickly identified due to the modal frequency and damping. Thus, the quantitative noise reduction effect of the improved scheme cannot be evaluated. The finite element simulation analysis method needs grids to be divided and various settings to be implemented, while the experimental analysis method needs sensors and debugging equipment to be arranged. The recognition efficiencies of these methods are lower. The effects of noise reduction effects were evaluated herein using simulation and equipment methods, and their efficiencies are also low. The energy amplification factor transmitted by the fan to the passive end of the cooling module can be quickly and accurately identified using the frequency difference sensitivity method. Consequently, the quantitative noise reduction effect of the improved scheme can be efficiently evaluated in advance.

## 5. Conclusions

The conventional modal frequency resonance method of obtaining modal frequency and damping cannot identify the energy amplification factor transmitted by the excitation source to the response end quickly and accurately as long as the excitation source is close to the natural frequency of the system. Consequently, the improvement effect cannot be further evaluated. The efficiency levels of the finite element simulation analysis and experimental analysis are also low. Therefore, a frequency difference sensitivity method was proposed in this paper, and can be used to identify and evaluate the resonance of the vehicle cooling module quickly and accurately. Our conclusions are as follows:

(1) The frequency difference sensitivity method proposed herein was applied to identify the resonant noise of a vehicle cooling module. A theoretical model for identifying the resonant noise amplification of a vehicle cooling module was established. The results show that the prediction error of this model is less than 5%.
(2) The frequency difference sensitivity method proposed herein was applied to evaluate the resonant noise of a vehicle cooling module. A theoretical model for evaluating the improved resonant noise reduction effect of a vehicle cooling module was established. The results show that the prediction error of this model is less than 5%.
(3) The interior noise amplified by the cooling module’s mode resonance was taken as an example to verify the high efficiency and practical effectiveness of the frequency difference sensitivity method proposed in this paper. Comparisons between the frequency difference sensitivity method and the conventional method were made. Our research shows that the energy amplification factor from the fan to the passive end of the cooling module can be predicted using the frequency difference sensitivity method more efficiently, meeting accuracy requirements. The quantized noise reduction effect achieved by the improvement scheme adopted herein can be predicted and evaluated more efficiently, also meeting accuracy requirements.

## Figures and Tables

**Table 1:** Theoretical model verification of response force with frequency difference by simulation and trial.

Modal Frequency (Hz)	Frequency Difference (Hz)	Frequency Difference (Normalization)	Response Force (Normalization)		
			Theory	Simulation	Trial
40	0	0	1.052	1	1
38.5	1.5	0.075	0.740	0.752	0.772

**Table 2:** Theoretical model verification of response force with frequency difference by simulation.

Scheme	Modal Frequency (Hz)	Frequency Difference (Hz)	Frequency Difference (Normalization)	Response Force (Normalization)	
				Theory	Simulation
Original state	40	0	0	1.052	1
Cut off bottom right vibration pad	38.5	1.5	0.075	0.740	0.752
Cut off bottom left vibration pad	37.5	2.5	0.125	0.570	0.585
Cut off top right vibration pad	36.5	3.5	0.175	0.431	0.452
Cut off top left vibration pad	35.5	4.5	0.225	0.322	0.345

**Table 3:** Comparison and verification of interior noise before and after the improvement scheme.

Scheme	Frequency Difference (Hz)	Frequency Difference (Normalization)	Noise Reduction (dB (A))		
			Theory	Simulation	Trial
Improvement effect	1.5	0.075	3.8	3.2	3.0

**Table 4:** Comparison and verification of interior noise before and after different improvement schemes.

Scheme	Frequency Difference (Hz)	Frequency Difference (Normalization)	Noise Reduction (dB (A))		
			Theory	Simulation	Trial
Cut off bottom right vibration pad	1.5	0.075	3.8	3.2	3.0
Cut off bottom left vibration pad	2.5	0.125	6.5	6.0	5.8
Cut off top right vibration pad	3.5	0.175	9.4	8.8	8.5
Cut off top left vibration pad	4.5	0.225	12.4	11.7	11.2

**Table 5:** Comparison of the Y-direction dynamic stiffness of vibration isolator before and after adjustment.

State Description	Y-Dynamic Stiffness (N/mm)		Natural Frequency of the Cooling Module (Hz)
	Top Vibration Pad	Bottom Vibration Pad	
Original state	135	157	40.1
After improvement	89	103	32.5

**Table 6:** Comparison of interior noise reduction at 32.5 Hz compared with 40 Hz (dB (A)).

Predicted Value of Single Fan	Measured Value of Single Fan Working	Test Value in Idle Conditions with Air Conditioning
19.0	19.6	19.4

## Data Availability

Data are contained within the article.
